# Which patient-specific and surgical characteristics influence postoperative pain after THA in a fast-track setting?

**DOI:** 10.1186/s12891-017-1725-8

**Published:** 2017-08-24

**Authors:** Yvon M. den Hartog, Gerjon Hannink, Nick T. van Dasselaar, Nina M. Mathijssen, Stephan B. Vehmeijer

**Affiliations:** 1 Department of Orthopaedic Surgery, Reinier de Graaf Hospital, Delft, the Netherlands; 2 Orthopaedic Research Lab, Department of Orthopaedics, Radboud university medical center, Nijmegen, the Netherlands; 3 Department of Anaesthesiology and Pain Medicine, Reinier de Graaf Hospital, Delft, the Netherlands

**Keywords:** Total hip arthroplasty, Fast-track, Multimodal pain protocol, Pain management, Postoperative pain

## Abstract

**Background:**

In our hospital a fast-track setting including a multimodal pain protocol is used for total hip arthroplasty (THA). Despite this multimodal pain protocol there is still a large range in reported postoperative pain between patients, which hinders mobilization and rehabilitation postoperatively. The goal of this study was to identify which patient-specific and surgical characteristics influence postoperative pain after THA in a fast-track setting.

**Methods:**

All 74 patients with osteoarthritis of the hip who underwent primary THA procedure by anterior supine intermuscular approach between November 2012 and January 2014 were included in this prospective cohort study. The protocol for pain medication was standardized.

Postoperative pain determined with the Numeric Rating Score was collected at 17 standardized moments. Linear mixed models were used to examine potential patient-specific and surgical factors associated with increased postoperative pain.

**Results:**

Pain patterns differed substantially across individuals. Adjusted for other variables in the model, preoperative use of pain medication (regression coefficient 0.78 (95% CI 0.28–1.26); *p* = 0.005) and preoperative neuropathic pain scored by DN4 (regression coefficient 0.68 (95% CI 0.15–1.20); *p* = 0.02) were the only factors significantly associated with higher postoperative pain scores.

**Conclusions:**

The knowledge of which factors are associated with higher postoperative pain scores after THA in a fast-track setting may help optimizing perioperative postoperative pain management and preoperative education of these patients.

**Trial registration:**

The study was retrospectively registered in the ISRCTN registry under identifier ISRCTN15422220 (date of registration: July 25, 2017).

## Background

Total hip arthroplasty (THA) is associated with considerable postoperative pain [1]. Almost all pain after surgery arises as a result of tissue damage at the surgical site [2]. This postoperative pain hinders early mobilization and rehabilitation with subsequent consequences on mobility and overall recovery [3]. In the last few years fast-track protocols have been introduced worldwide for elective primary THA. These are partly based on pain management protocols and include a rigorous perioperative pain management program, which allow for an optimized perioperative period [4, 5]. In 2009, a fast-track protocol including a multimodal pain protocol was successfully introduced in our teaching hospital [6]. The multimodal pain protocol, developed to reduce acute postoperative pain to enable quick mobilization and rehabilitation included paracetamol, celecoxib, gabapentine, dexamethasone and esketamine [7–12]. Furthermore, we use the anterior supine intermuscular (ASI) approach for THA procedures in our hospital. Since this approach uses both an intermuscular and internervous plane and causes less surgical trauma, lower postoperative pain scores and less pain medication consumption have been described for this approach [13–18]. Despite the introduction of a multimodal pain protocol and use of the ASI approach, there is still a large range in reported postoperative pain between patients.

Previous studies have shown that specific patient- and provider characteristics could influence postoperative pain [19–27]. Only one of these studies reported solely on postoperative pain after primary THA [21]. None of these studies were performed in a fast-track setting and no multimodal pain protocols developed to reduce acute postoperative pain were included in these studies.

The proper identification of patients who are at risk to experience more postoperative pain directly after primary THA might provide details for further optimization of postoperative pain management and preoperative education of these patients. Therefore, the goal of this study was to identify which patient-specific and surgical characteristics influence postoperative pain after primary THA by ASI approach in a fast-track setting including a multimodal pain protocol.

## Methods

All 74 patients with osteoarthritis (72 patients with primary osteoarthritis, 2 patients with secondary osteoarthritis) of the hip who underwent primary THA procedure by ASI approach between November 2012 and January 2014 were included in this prospective cohort study. All procedures were performed in a fast-track setting, by one experienced orthopedic hip surgeon (SBV). Patients with neurological conditions which potentially influence pain perception; American Society of Anaesthesiologists (ASA) classification III/IV; medical contra-indication for spinal anesthesia; cardiovascular impairment in the present or past; known allergy against any element of the medication that is given for the multimodal pain protocol; abuse of alcohol or drugs; rheumatoid arthritis; BMI > 40 kg/m^2^; and patients with cognitive impairment were excluded.

As part of the fast-track setting, all patients joined a patient education program prior to surgery. All patients received spinal anesthesia with a low dose of bupivacaine (6–8 mg intrathecally). Propofol was administered for sedation and to allow a single shot of esketamine. The multimodal protocol for perioperative pain medication was standardized (Table 1). Before discharge, adequate pain relief had to be achieved by oral pain medication: the Numeric Rating Scale (NRS) for pain had to be below 3 in rest and below 5 during mobilization (NRS; 0 to 10, best to worst).

**Table 1:** The standardized multimodal protocol for perioperative pain medication

Timing	Medication
2 h before surgery	Paracetamol (acetaminophen) 1000 mg per os.
	Celecoxib (Celebrex®) ^a^ 400 mg per os.
	Gabapentin 600 mg per os.
Just before surgery	Dexamethasone 0.15 mg/kg iv. ^b^
	Esketamine 15 mg iv.
4 h after surgery	Paracetamol (acetaminophen) 1000 mg per os.
8 h after surgery	Paracetamol (acetaminophen) 1000 mg per os.
	Gabapentin 300 mg per os.
Before the night	Tramadol 100 mg supp.
Day 1	Paracetamol (acetaminophen) 1000 mg per os 4 times a day.
	Celecoxib (Celebrex®^) a^ 200 mg per os in the morning.
	Gabapentin 300 mg per os in the morning.
After day 1	Paracetamol (acetaminophen) 1000 mg per os 4 times a day (with a maximum of 2 weeks).
	Celecoxib (Celebrex®) ^a^ 200 mg per os in the morning (until 2 weeks after surgery).
Rescue medication	Celecoxib (Celebrex®) ^a^ 200 mg per os extra after the first night Piritramid (Dipidolor®) 10 mg im, which could be repeated every 4 h.

The standardized multimodal protocol for perioperative pain medication was determined based on literature [7–12]

^a^In combination with celecoxib (Celebrex®) all patients will receive omeprazol 20 mg per os once a day as prophylaxis. When the patient was already using a proton pomp inhibitor before admittance no omeprazol was administered

^b^Dexamethasone solution in 50cm^3^ saline is administered slowly to avoid adverse side effects like severe perianal pain

**Fig. 1:**
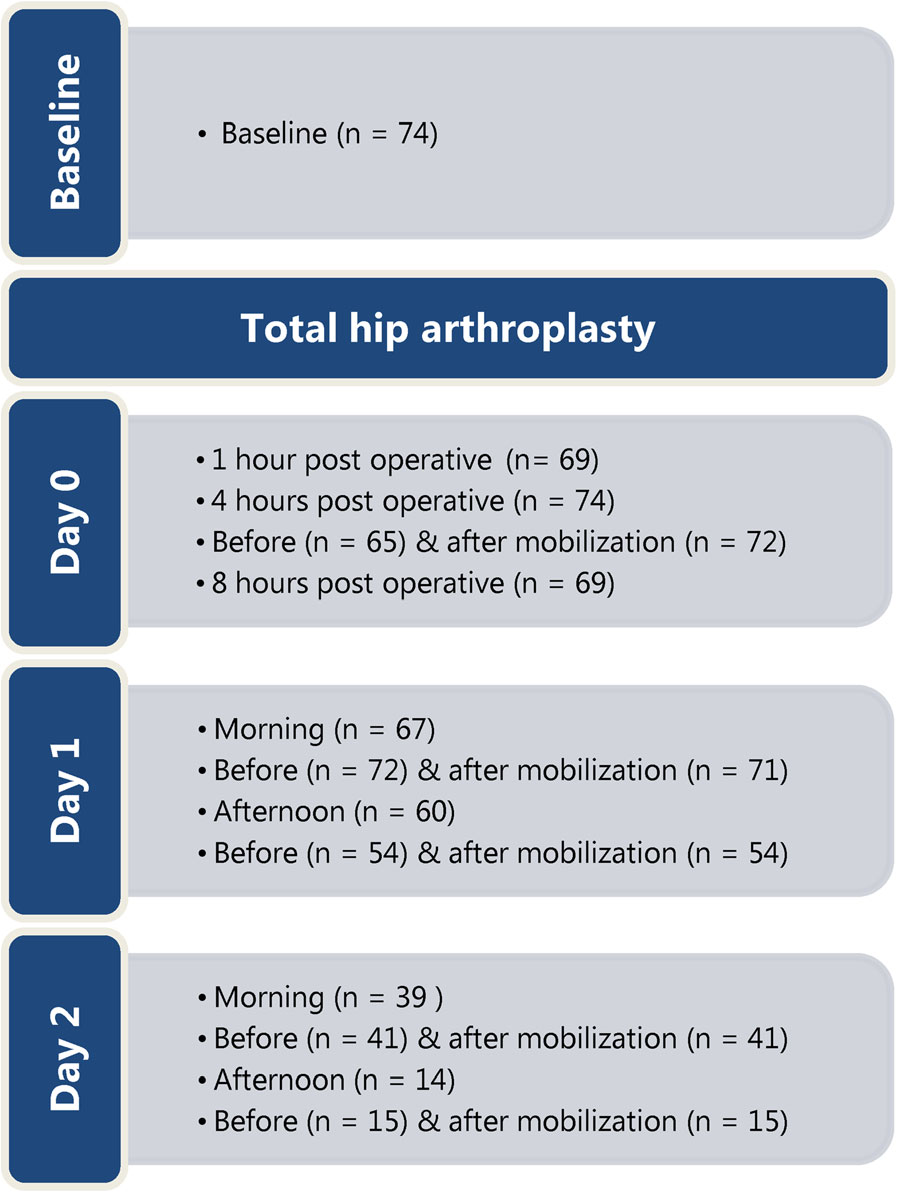
Overview of pre- and postoperative pain measurements. Postoperative pain determined with the NRS was collected at 17 standardised moments, from 1 h after surgery until the afternoon of the second day after surgery

Postoperative pain determined with the NRS was collected at 17 standardized moments, from 1 h after surgery until the afternoon of the second day after surgery (Fig. 1). The study protocol was approved by our local Ethics Committee (2012–000989-37/NL39970.098.12). The study was retrospectively registered in the ISRCTN registry under identifier ISRCTN15422220 (date of registration: July 25, 2017).

Potential factors associated with increased postoperative pain (gender; ASA classification; age; BMI; diabetes mellitus (DM); surgery time; incision length; living situation; preoperative pain determined with the NRS, preoperative use of pain medication; use of antidepressants; as well as preoperative scores of the neuropathic pain diagnostic questionnaire (DN4), and Amsterdam Preoperative Anxiety and Information Scale (APAIS) for anxiety and information requirements) were examined with univariable linear mixed models for repeated measures. Decision to include these variables was based on guidance from directed acyclic graphs [28]. Based on the criteria in the original article about the APAIS by Moerman and others [29]*,* the APAIS anxiety scale was dichotomized (4–10: no, 11–20: yes), and the APAIS need-for-information scale was divided into three categories (2–4: no or little, 5–7: average, and 8–10: high). The DN4 questionnaire score was dichotomized (1–3 unlikely; 4–7 likely) [30, 31].

Factors that were associated with the outcome in univariable analyses (*p*-values <0.20) were included in multivariable analyses. In the multivariable analyses *p*-values less than 0.05 were considered significant. Missing data were assumed to be missing at random. Regression coefficients are presented with their 95% confidence intervals. The statistical analyses were performed using R version 3.1.2 with package ‘nlme’ [32, 33].

## Results

Mean age of all patients was 67.1 year (range 42.7–84.6) and mean BMI was 27.1 kg/m^2^ (range 20.1–38.9). Mean LOS was 1.8 nights (range 1–7). Most patients lived together with cohabitants (*n* = 58; 78.4%) and were discharged to their own home (*n* = 72; 97.3%). Patient characteristics are listed in Table 2.

**Table 2:** Patient characteristics for the total group of 74 patients undergoing primary total hip arthroplasty

		Total n (%) *n* = 74
Age (year)		67.1 (42.7–84.6) ^a^
BMI (kg/m^2^)		27.1 (20.1–38.9) ^a^
Gender	male	36 (48.6%)
ASA classification	ASA2	47 (63.5%)
Surgery time (minutes)		79.2 (49–116) ^a^
Diabetes Mellitus		7 (9.5%)
Incision length (cm)		9.97 (7.5–12.0) ^a^
Living situation	with cohabitants	58 (78.4%)
Preoperative antidepressants use		4 (5.4%)
Preoperative pain medication use		50 (67.6%)
Preoperative pain (NRS) ^b^		5.26 (0–9) ^a^
DN4	likely	16 (21.6%)
APAIS anxiety	yes	15 (23.1%)
APAIS information	no/little	24 (37.5%)
	average	24 (37.5%)
	high	16 (25.0%)

^a^mean (range)

^b^ *n* = 72 patients

Seventy-three patients received an uncemented prostheses (Taperloc® femoral prosthesis and an Universal® cup, both Biomet, Warsaw, In, USA), one patient received a cemented prosthesis because of inferior bone quality due to severe osteoporosis. (Exceed Muller® cup and a Taperloc® femoral prosthesis, both Biomet, Warsaw, In, USA).

Pain patterns differed substantially across individuals. Moreover, pain varied across the standardized moments (Table 3). Adjusted for the other factors in the model, preoperative use of pain medication (regression coefficient 0.78 (95% CI 0.28–1.26); *p* = 0.005) and preoperative neuropathic pain scored by DN4 (regression coefficient 0.68 (95% CI 0.15–1.20); *p* = 0.02) were the only factors that were significantly associated with higher postoperative pain scores (Table 4).

**Table 3:** Pain scores determined with the NRS at the standardized moments

NRS		N	mean (range)
Preoperative		72	5.26 (0.0–9.0)
Day of surgery	1 h	69	1.51 (0.0–8.0)
	4 h	74	2.97 (0.0–9.0)
	Before mobilization	69	2.62 (0.0–6.0)
	After mobilization	65	2.93 (0.0–10.0)
	8 h	72	2.40 (0.0–8.0)
Day 1	Morning	67	1.63 (0.0–5.0)
	Before mobilization	72	1.71 (0.0–7.0)
	After mobilization	71	2.25 (0.0–9.0)
	Afternoon	60	1.57 (0.0–6.0)
	Before mobilization	54	1.26 (0.0–4.0)
	After mobilization	54	1.73 (0.0–6.0)
Day 2	Morning	39	2.00 (0.0–7.0)
	Before mobilization	41	1.54 (0.0–6.0)
	After mobilization	41	1.84 (0.0–4.5)
	Afternoon	14	1.82 (0.0–3.0)
	Before mobilization	15	1.27 (0.0–3.0)
	After mobilization	15	1.93 (0.0–4.0)

**Table 4:** Regression coefficients with 95% CIs for potential factors associated with increased postoperative pain after THA in a fast-track setting

		Univariable analyses		Multivariable analysis	
		coefficient (95% CI)	*p*-value	coefficient (95% CI)	*p*-value
Age		0.006 (−0.02–0.03)	0.66	–	–
BMI		0.03 (−0.03–0.09)	0.28	–	–
Gender	male vs. female	−0.31 (−0.79–0.18)	0.22	–	–
ASA classification	ASA2 vs. ASA1	0.30 (−0.21–0.80)	0.25	–	–
Surgery time		−0.01 (−0.03–0.0005)	0.06	0.0004 (−0.01–0.01)	0.96
Diabetes Mellitus		−0.05 (−0.88–0.78)	0.92	–	–
Incision length		0.02 (−0.26–0.31)	0.89	–	–
Living situation	with cohabitants vs. alone	0.53 (−0.05–1.10)	0.08	0.50 (−0.08–1.07)	0.11
Preoperative antidepressants use	yes vs. no	0.66 (−0.39–1.71)	0.22	–	–
Preoperative pain medication use	yes vs. no	0.68 (0.18–1.18)	0.009	0.78 (0.28–1.26)	0.005
Preoperative pain		0.10 (−0.01–0.21)	0.08	−0.02 (−0.13–0.09)	0.73
DN4	likely vs. unlikely	0.70 (0.13–1.28)	0.02	0.68 (0.15–1.20)	0.02
APAIS anxiety	yes vs. no	−0.06 (−0.67–0.54)	0.84	–	–
APAIS information	average vs. no/little	−0.02 (−0.61–0.56)	0.93	−0.21 (−0.74–0.31)	0.45
	high vs. no/little	0.50 (−0.16–1.15)	0.15	0.45 (−0.12–1.02)	0.15

Factors that were associated with the outcome in univariable analyses (*p*-values <0.20) were included in a multivariable linear mixed model for repeated measures. In the multivariable analyses *p*-values <0.05 were considered significant

## Discussion

The aim of this study was to identify which patient-specific and surgical characteristics influence postoperative pain after primary THA by ASI approach in a fast-track setting, using a multimodal pain protocol which was developed to reduce acute postoperative pain to enable quick mobilization and rehabilitation. The only two factors associated with increased postoperative pain adjusted for the other factors in the model, were preoperative use of pain medication (regression coefficient 0.78 (95% CI 0.28–1.26); *p* = 0.005) and preoperative neuropathic pain scored by DN4 (regression coefficient 0.68 (95% CI 0.15–1.20); *p* = 0.02).

In our study, we used a multimodal pain protocol, including paracetamol, celecoxib, gabapentin, dexamethasone and esketamine [7–12]. This pain protocol is part of a fast-track setting and is developed to reduce acute postoperative pain to enable patients to quickly mobilize and rehabilitate in an optimized and safe perioperative period [4, 5]. Mean postoperative pain score determined with the NRS collected at 17 standardized moments, varied from 1.51 (range 0.0–8.0) to 2.97 (range 0.0–9.0). These results demonstrate that the use of our multimodal pain protocol enables adequate postoperative pain relief in which patients are able to mobilize and rehabilitate quickly. However, despite this pain protocol, a large range in reported postoperative pain between patients still exists, including some outliers with high postoperative pain scores.

Pain has been described to be a sensory and emotional experience that is influenced by multiple factors [34, 35]. Although several other studies reported on effects of specific patient- and provider characteristics on postoperative pain [19–27], none of these studies were performed in a fast-track setting with a multimodal pain protocols developed to reduce acute postoperative pain (and hence enable early mobilization), were included in these studies. Since no literature reports on the effect of potential factors associated with increased postoperative pain in a fast-track setting, our model included various potential factors that have been described to be associated with postoperative pain based on a non-fast-track setting [19–27, 36]. The use of a multimodal pain protocol in our study might have influenced the effects of these characteristics on postoperative pain and could be a reason for discrepancy between our results and results of these other studies.

Preoperative use of pain medication provides information on the preoperative pain levels of patients and has been shown to be associated with more severe postoperative pain [26, 27]. Our study supports these findings. In contrast, another study on postoperative pain 12 to 24 h after elective abdominal surgery, demonstrated no effect of preoperative use of pain medication on postoperative pain [23].

Our multimodal protocol for postoperative pain medication was standardized for all patients. The effect of this pain protocol might therefore not be sufficient for single patients who are used to pain medication. On the other hand, in a study on postoperative pain after thoracic surgery a decrease in postoperative pain medication use for patients who used pain medication preoperatively was found [37].

DN4 [30, 31] is a validated questionnaire for neuropathic pain, which was preoperatively scored for all patients in our study. The differences between neuropathic pain and non-neuropathic (nociceptive) pain have been described in literature [30]. Osteoarthritis, the main indication for THA in our study, causes non-neuropathic pain [30]. Patients who experience non-neuropathic pain from osteoarthritis preoperatively, are more likely to benefit from THA and will experience less pain postoperatively. Since preoperative neuropathic pain is not caused by osteoarthritis of the hip [30], THA will probably not resolve this neuropathic pain. As a consequence, these patients will be more likely to experience more postoperative pain after primary THA.

In the present study, none of the other factors were significantly associated with postoperative pain. All of these potential factors have been described to be associated with postoperative pain in non-fast-track setting studies [19–27, 36]. Contrasting results on the effect of age on postoperative pain have been reported in literature, including an association between younger age and a higher level of postoperative pain [20, 23, 24, 27] as well as a lack of effect of age on the level of postoperative pain [26, 38].

BMI has been shown to be associated with an increased inflammatory response [38], which is related to higher levels of postoperative pain [39], whereas others demonstrated no effect of BMI on postoperative pain scores [24, 39, 40]. In our study we excluded patients with BMI > 40 kg/m^2^, which might be a reason for lack of effect of BMI on postoperative pain.

Regarding gender, contrasting results on the effect on postoperative pain have been reported in literature as well. These results include both higher postoperative pain for female patients [24, 27, 38] and no effect of gender on postoperative pain [22, 23, 25, 26].

Higher ASA classification has also been shown to be associated with increased postoperative pain [23]. However, in our study we only included patients with ASA classification I or II, which might be a reason for lack of effect of ASA classification on postoperative pain.

An increased incision length results in increased tissue damage, and might subsequently result in increased postoperative pain. This relation has been described in literature [24]. In our study the ASI approach was used. Since this approach uses both an intermuscular and internervous plane, less surgical trauma and hence lower postoperative pain scores and less pain medication consumption have been described [13–18].

Use of antidepressants provides information on patients’ mental state, which could be predictive for the patients’ response on pain medication and pain experience [25]. Moreover, in literature depression symptoms have been mentioned to be related to higher level of postoperative pain [23, 38, 41].

Furthermore, we used different validated questionnaires preoperatively for all patients to define preoperative pain and pain characteristics, including the APAIS [29]. Anxious patients respond differently to anesthesia and pain than non-anxious patients and therefore require larger doses of anaesthetics [19, 20, 25, 42, 43]. It has been reported that the anxiety/‘worry’ component of the APAIS is positively associated with the occurrence of early postoperative pain, whereas a strong information seeking behavior reduces the incidence of severe postoperative pain [24]. This is in contrast to others who report that patients who require more information about impending discomforts preoperatively may sensitize the individual to the experience [19].

Some potential limitations of our study should be discussed. First, although only 74 patients were included in the present study, a strength of the study is that we used linear mixed models. These do not only model the correlation between repeated measures of the same patient, they also assess fluctuations in postoperative pain over time. Second, although others investigated effects of occupation and/or level of education [19, 23, 40, 41], SF-36 [24], and heart rate and blood pressure [36] on postoperative pain, we were not able to include these variables in our model, because these variables were not reported in a consistent way in the patient files. Third, one single orthopaedic surgeon performed all surgical procedures which reduces variation in operative technique, however, could potentially mask systematic faults in surgical technique or surgical approach. Fourth, the indication of THA could be questioned in patients who do not need any preoperative pain medication. In our study, 50 out of 76 reported to use preoperative pain medication. National and international evidence-based guidelines for hip and knee OA recommend to start with (a combination of) conservative treatments. Despite the recommendation in guidelines to start with conservative treatments and only use surgical intervention if a patient does not respond sufficiently to conservative treatment options, the use of conservative treatments in daily practice is suboptimal [44–47]. It has been shown that conservative treatments were not fully exploited in 81% of the patients who were referred to specialized knee/hip OA outpatient clinics [44]. Even worse, a recent study showed that only 10% of the patients in orthopaedic practice received all recommended non-surgical treatments before surgery [48].

A qualitative systematic review of Ip and others [36] identified factors associated with postoperative pain and analgesic consumption. Type of surgery was an important predictive factor for postoperative pain. This suggests that results of our study are applicable for primary THA. Furthermore, this could be another reason for the discrepancy between different studies describing postoperative pain after different types of surgery, besides differences in the pain protocol used.

## Conclusion

Only preoperative use of pain medication and preoperative neuropathic pain were associated with increased postoperative pain after primary THA in a fast-track setting, including a multimodal pain protocol which was developed to reduce acute postoperative pain to enable quick mobilization and rehabilitation. This knowledge provides further details for optimization of postoperative pain management and preoperative patient education.

## Abbreviations

APAIS: Amsterdam preoperative anxiety and information scale
ASA: American Society of Anaesthesiologists
ASI: Anterior supine intermuscular
BMI: Body mass index
DM: Diabetes mellitus
DN4: Douleur neuropathique en 4 questions
LOS: Length of hospital stay
NRS: Numeric rating scale
THA: Total hip arthroplasty
